# Incremental Value of Non‐Gated Chest CT Coronary Artery Calcium Score in Predicting Major Adverse Cardiovascular Events by GRACE Score After Percutaneous Coronary Intervention in Patients With Acute Coronary Syndrome

**DOI:** 10.1002/clc.70242

**Published:** 2025-12-29

**Authors:** Zhaoyuan Xing, Haoyan Pan, Huan Ding, Jianing Chen, ZiGuang Huang, Jing Wen, Zhe Zhang, Baoying Zhao, Xu Dai

**Affiliations:** ^1^ Liaoning University of Traditional Chinese Medicine Shenyang Liaoning China; ^2^ CT Clinical Science, Philips Healthcare Shenyang China; ^3^ Affiliated Hospital of Liaoning University of Traditional Chinese Medicine Shenyang Liaoning China

**Keywords:** ACS, GRACE risk score, MACE, non‐gated chest CT coronary artery calcification score, PCI

## Abstract

**Objective:**

To evaluate the incremental value of non‐gated chest CT coronary artery calcium score in enhancing GRACE score prediction of major adverse cardiovascular events (MACE) after percutaneous coronary intervention (PCI) in patients with acute coronary syndrome (ACS).

**Methods:**

A retrospective cohort study was conducted on 324 ACS patients undergoing PCI and non‐gated chest CT. Patients were divided into MACE (*n* = 100) and non‐MACE (*n* = 224) groups with a median follow‐up of 18.7 months. The predictive performance of the GRACE score, Agatston score, and combined clinical composite model was evaluated using receiver operating characteristic (ROC) curves and survival analysis based on optimal cutoff values.

**Results:**

Model 3 (GRACE + CACS) demonstrated AUC values of 0.798 and 0.827 in the training and testing cohorts, respectively, significantly outperforming Model 1 (GRACE) (training AUC = 0.702; testing AUC = 0.758). Model 4, incorporating clinical features, demonstrated optimal predictive performance (training set AUC = 0.806; testing set AUC = 0.857). The AUC differences were statistically significant (*p* < 0.05). Survival curves revealed the highest MACE incidence (94.4%, *p* < 0.01) in the high‐risk combined Ga1 group (GRACE ≥ 140 and Agatston ≥ 400).

**Conclusion:**

The non‐gated chest CT coronary calcification score significantly enhances the predictive value of the GRACE score for major adverse coronary events (MACE) after coronary intervention. When combined with clinical indicators, the predictive power is further improved. Sensitivity analysis confirms the robustness of this finding, providing a reliable tool for clinical risk stratification.

## Graphical abstract

This study confirms that the combined GRACE score and coronary artery calcium score significantly improves the predictive efficacy of the risk of major adverse cardiovascular events (MACE) after percutaneous coronary intervention (PCI) in patients with acute coronary syndrome (ACS), and the predictive value is even better when combined with clinical characteristics.

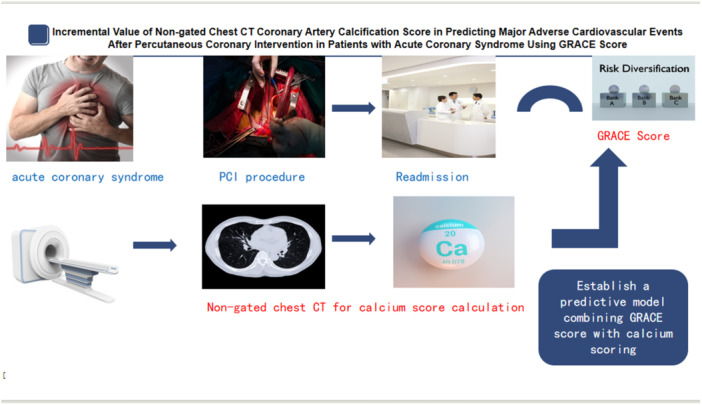

Acute coronary syndrome (ACS), a cardiovascular emergency, arises from the pathophysiological instability of atherosclerotic plaques (including rupture or erosion), leading to coronary thrombosis and ultimately triggering a clinical syndrome characterized by acute myocardial ischemia [1]. As the core reperfusion strategy for ACS, percutaneous coronary intervention (PCI) rapidly restores blood flow through lesion vessels via balloon dilatation and stent implantation. Early hemodilution has been demonstrated to play a crucial role in preserving myocardial tissue and improving outcomes in ACS patients [2, 3]. However, post‐PCI pharmacotherapy and reperfusion therapy remain underutilized [4]. A 4‐year nationwide database analysis in the United States revealed that approximately one‐quarter of PCI patients experienced unplanned readmission within 6 months, with cardiac‐related causes constituting the primary factor [5]. Early readmission after PCI (incidence 2.5%–9.8%) imposes a significant economic and health burden: annual costs reach $26 billion. Studies confirm readmission substantially impairs patients′ quality of life and health status, leading to worsening disease severity over time [6].

The most widely used acute coronary syndrome risk score in clinical practice is the GRACE score for acute coronary events. GRACE, an international registry for ACS patients, developed the GRACE risk score to predict mortality. The GRACE score effectively determines the probability of patients experiencing MACE in the near and long term [7]. It is now also frequently used as a predictive tool for the incidence of MACE following PCI in ACS patients [8]. Although the GRACE score provides important prognostic information, its reliance on primarily clinical and biochemical parameters may not fully reflect underlying coronary atherosclerotic burden. Coronary artery calcium (CAC), a recognized marker of atherosclerosis, may offer incremental prognostic value when combined with clinical risk scores [9]. Therefore, exploring the additional predictive utility of CAC scoring in ACS patients undergoing PCI holds significant clinical importance [9]. Calcification scores (CACS) serve as independent predictors and indicators of coronary atherosclerosis severity and cardiovascular risk. These scores quantify the degree of atherosclerosis in the vascular wall by measuring the quantity and density of calcified plaques within the coronary arteries [10]. Coronary artery calcification scoring can be performed using the Agatston score, volumetric score, or mass score, with the Agatston score being the most commonly used [11, 12]. Studies have shown that the Agatston score is an independent predictor of MACE in patients after PCI [13], and have found a positive correlation between the GRACE score and the Agatston score. Currently, traditional chest CT calcium scores can replace coronary CT angiography calcium scores, addressing the drawbacks of coronary CT angiography such as high radiation exposure, contrast agent injection, repeated scans, and high costs [14]. This study innovatively integrated the GRACE clinical risk score with non‐gated chest CT coronary calcification scores and clinical indicators to construct a multidimensional combined prediction model, aiming to significantly improve the predictive accuracy of MACE in ACS patients after PCI.

## 1 Methods

### 1.1 Study Population

**Figure 1:**
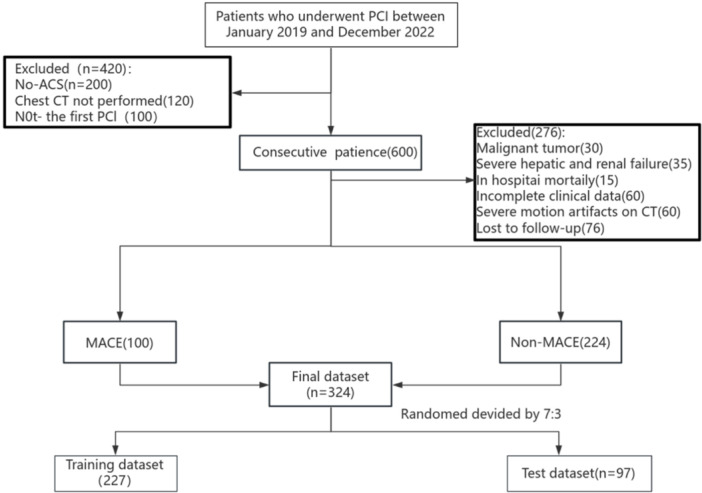
Patient recruitment and study design flowchart. ACS, acute coronary syndrome; PCI, percutaneous coronary intervention; MACE, major adverse cardiovascular events.

Clinical data were retrospectively collected from 324 patients who underwent PCI for ACS and concurrent routine chest CT examinations at the Department of Cardiovascular Medicine, Liaoning University of Traditional Chinese Medicine Affiliated Hospital between January 2019 and December 2022. Patients were categorized into a MACE group (*n* = 100) and a non‐MACE group (*n* = 224) based on MACE occurrence post‐discharge. MACE is defined as any of the following events occurring for the first time during post‐discharge follow‐up: cardiac death (including fatal acute myocardial infarction), non‐fatal acute myocardial infarction, malignant arrhythmia, any unplanned coronary revascularization, readmission due to heart failure, and non‐fatal stroke [15] (Figure 1). All enrolled patients were randomly assigned to the training set (*n* = 146) and test set (*n* = 38) in a 7:3 ratio. To assess the robustness of endpoint definitions, sensitivity analyses were conducted by redefining the primary endpoint MACE as an event combination excluding “readmission due to heart failure.” This study was reviewed and approved by the hospital ethics committee [NO.Y2025005CS(KT)‐005‐01].

Inclusion criteria: The study cohort included all patients who underwent initial PCI for ACS between 2019 and 2022 and survived to hospital discharge. Exclusion criteria: (1) Malignant tumors with severe infection (30 cases); (2) Severe hepatic failure and end‐stage renal disease (35 cases); (3) In‐hospital death (15 cases); (4) Incomplete clinical data (60 cases); (5) Severe motion artifacts on chest CT (60 cases); (6) Failure to follow up (76 cases). (Sample size calculation formula: n_events ≥ m × EPV, where m is the number of predictor variables in the model and EPV is the number of events per variable)

Clinical Data Collection: Based on the hospital's electronic medical record system, the following patient history data and diagnostic information were collected: (1) Available clinical risk factors for each patient, including cardiovascular risk factors, medication history, and biochemical indicators. (2) GRACE score at admission: Scored based on age, heart rate, systolic blood pressure, creatinine, Killip class, cardiac arrest at admission, elevated cardiac biomarkers, and ST‐segment deviation. Risk stratification as follows: Low risk: (≤ 108 points); Moderate risk: (109–140 points); High risk: (> 140 points).

### 1.2 Conventional Chest Plain CT

All non‐gated chest CT examinations in this study were performed as part of the hospital's standard preoperative evaluation protocol following patient admission for ACS. The primary objectives were to rule out pulmonary complications, assess mediastinal conditions, and aid in the differential diagnosis of chest pain. All CT examinations were completed prior to the initial PCI procedure. Scans were performed using 256‐slice spiral CT (Brilliance iCT, Philips, Netherlands), 128‐slice spiral CT (Neuviz, Neusoft Group, China), (Brilliance, Philips, Netherlands), or (Incisive, Philips, Netherlands). Patients positioned supine with head‐first insertion. Scanning range: From the thoracic inlet to the cardiac diaphragmatic plane. Scan parameters: Tube voltage 120 kV, tube current automatically modulated, gantry rotation speed 0.5 s/rev, gantry rotation time 1, matrix 512 × 512, slice thickness and interval 1 mm. All CT images were transferred to a post‐processing workstation (IntelliSpace Portal Vision 9.0 Philips Healthcare, Best, The Netherlands) and independently evaluated and analyzed by two experienced cardiovascular radiologists.

### 1.3 Image Data Acquisition

**Figure 2:**
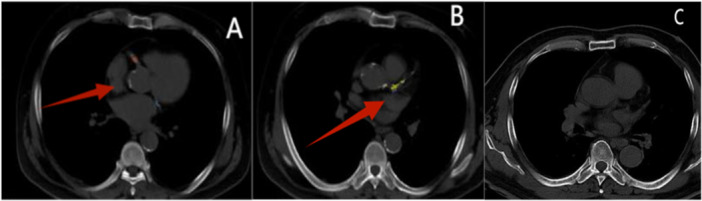
(A) MACE patient, male, 43 years old, unstable angina, follow‐up after MACE (underwent unplanned revascularization 10 months later). GRACE score: 162 (high risk) Agatston score: 6276 (high risk). (B) MACE patient, male, 67 years old, acute coronary syndrome, follow‐up after MACE (unplanned revascularization at 18 months). GRACE score: 115 (intermediate risk) Agatston score: 1784 (high risk). (C) Non‐MACE patient, male, 67 years old, unstable angina, follow‐up without MACE (after 50 months). GRACE score: 136 Moderate risk Agatston score: 0 Very low risk.

Quantitative analysis of coronary artery calcification was performed using an artificial intelligence analysis system developed by Shukun Technology Co. Ltd., which enables automated processing of non‐gated chest CT image data. We utilized an artificial intelligence analysis system developed by Shukun Technology Co. Ltd. to perform automated calcium scoring on non‐gated chest CT images. This system, based on a three‐dimensional Unet neural network architecture, enables automatic identification and segmentation of coronary artery calcified plaques from raw image data. The specific workflow includes: identification and three‐dimensional segmentation of calcified lesions in the left main, left anterior descending, left circumflex, and right coronary arteries, respectively. Following the standard Agatston calculation method, with threshold criteria of calcified region pixel area ≥ 1 mm² and CT value ≥ 130 HU, the Agatston score, equivalent calcium mass, and calcium volume score were calculated for each vascular segment, which were then aggregated into the total coronary artery calcium score. Following the American College of Cardiology/American Heart Association (ACC/AHA) guidelines [13] (Figure 2), Agatston scores were categorized into three risk levels—low‐risk (1–100 AU), intermediate‐risk (101–400 AU), and high‐risk (> 400 AU)—based on these thresholds.

### 1.4 Statistical Analysis

Data were analyzed using SPSS 26.0 and R software. Categorical data are presented as frequencies and percentages, with intergroup comparisons performed using chi‐square tests. For normally distributed continuous variables, data are expressed as mean ± standard deviation (x ± s) and evaluated for intergroup differences using independent samples t‐tests. For non‐normally distributed measurements, trends and dispersion are represented using median and interquartile range (M(P25,P75)). Intergroup comparisons for non‐normally distributed measurements were performed using the Mann‐Whitney U test. Additionally, Spearman correlation analysis was conducted to assess relationships between variables. Multivariate Cox regression analysis was performed on variables significant in univariate analysis to identify independent predictors of MACE. Receiver operating characteristic (ROC) curves were plotted for GRACE scores, calcification scores, and composite scores, The area under the curve (AUC) was analyzed for all three methods. Survival curves were plotted using the Kaplan‐Meier method and analyzed with the Log‐rank test. A *p* value < 0.05 was considered statistically significant. In sensitivity analysis, we used the exact same multivariate Cox proportional hazards model as the primary analysis to evaluate the association between all included predictive variables (including Agatston score and clinical risk factors) and the redefined MACE endpoint.

## 2 Results

### 2.1 Impact of GRACE Score and Calcification Score

Spearman′s rank correlation analysis revealed significant positive correlations between GRACE scores and Agatston scores with MACE in both the training set (r_G_= 0.327; r_a_ = 0.476) and the test set (r_G_= 0.343; r_a_ = 0.457), with statistically significant differences (*p* < 0.05). See Table 1.

**Table 1:** Correlation analysis between GRACE scores, calcification scores, and the incidence of adverse cardiovascular events after PCI in ACS patients.

				GRACE score	Volume integral	Equivalent mass	Agaston score
Spearman, Rho	MACE	Correlation Coefficient	Training group	0.327**	0.431**	0.472**	0.476**
		Sig. (bobtail)		0.000	0.000	0.000	0.000
		Correlation Coefficient	Test Group	0.343**	0.421**	0.460**	0.457**
		Sig. (bobtail)		0.000	0.000	0.000	0.000
**Significantly correlated at the 0.01 level (two‐tailed).							

** *p* < 0.01.

### 2.2 Clinical Data Comparison Between MACE and Non‐MACE Patients

Among patients with MACE, the following components were observed: heart failure in eight patients (8%), cardiovascular death in four patients (4%), malignant arrhythmia in nine patients (9%), non‐fatal myocardial infarction in two patients (2%), unplanned revascularization in 65 patients (65%), and stroke in 12 patients (12%). In both the training and validation cohorts, patients with and without MACE were well‐matched for gender, cardiovascular risk factors, and medication use, with no significant differences between groups (*p* > 0.05). A statistically significant difference in lipoprotein(a) levels was observed between the two cohorts (*p* < 0.05). Detailed clinical data comparisons are presented in Table 2.

**Table 2:** Comparison of clinical data between groups [(X ± s), M(P25,P75), n(%)].

	Training group			Test group		
	MACE group (*n* = 74)	Non‐MACE group (*n* = 153)	*p* value	MACE group (*n* = 26)	Non‐MACE group (*n* = 71)	*p* value
Cardiovascular risk factors						
Gender, male	50 (67.57)	95 (62.09)	0.422	15 (57.69)	41 (57.75)	0.996
Smoking	32 (43.24)	47 (30.72)	0.064	8 (30.77)	23 (32.39)	0.880
Alcohol consumption	14 (18.9)	29 (18.95)	0.977	4 (15.38)	7 (9.86)	0.449
Family History	5 (6.76)	13 (8.50)	0.650	2 (7.69)	8 (11.27)	0.610
Hypertension	45 (60.81)	93 (60.78)	0.997	18 (69.23)	39 (54.93)	0.207
Diabetes	22 (29.73)	37 (24.18)	0.880	6 (23.08)	19 (26.76)	0.715
Drug						
Anti‐arrhythmic	24 (32.43)	32 (20.92)	0.060	9 (34.6)	15 (21.13)	0.175
lood Pressure Control	15 (20.27)	33 (21.57)	0.823	10 (38.46)	18 (25.35)	0.209
Blood Glucose Control	23 (31.08)	51 (33.33)	0.735	7 (26.92)	22 (30.99)	0.700
Lipid Regulation & Pigmentation Control	56 (75.68)	118 (77.12)	0.809	20 (76.92)	52 (73.24)	0.715
Antiplatelet	71 (95.95)	138 (90.20)	0.134	26 (100.00)	66 (92.96)	0.167
Anticoagulant	3 (4.05)	3 (1.96)	0.358	0 (0.00)	1 (1.41)	0.545
Clinical Biochemical Test Indicators						
Total cholesterol, mmol/L	4.37 (3.35, 5.29)	4.36 (3.49, 5.30)	0.753	4.55 (3.43, 5.52)	4.23 (3.34, 5.79)	0.864
Triglycerides, mmol/L	1.51 (1.04, 2.39)	1.55 (1.21, 2.24)	0.342	1.51 (1.16, 2.07)	1.50 (1.07, 2.30)	1.000
High‐density lipoprotein cholesterol, mmol/L	1.04 (0.94, 1.31)	1.08 (0.94, 1.42)	0.120	1.10 (0.88, 1.32)	1.22 (0.95, 1.49)	0.228
Low‐density lipoprotein cholesterol, mmol/L	2.77 (2.08, 3.64)	2.80 (2.19, 3.68)	0.742	2.56 (2.22, 3.60)	2.65 (2.07, 3.81)	0.717
Blood glucose, mmol/L	6.61 (5.39, 8.69)	6.85 (5.78, 8.80)	0.173	56.85 (5.45, 9.05)	6.81 (5.39, 8.79)	0.893
C‐reactive protein, mmol/L	4.40 (2.76, 7.86)	4.40 (1.28, 6.10)	0.666	4.65 (0.99, 9.91)	4.45 (3.30, 5.70)	0.763
Lipoprotein a, mmol/L	18.85 (7.75, 30.23)*	12.20 (6.90, 22.55)*	0.033	19.40 (12.85, 27.05)**	11.65 (5.13, 16.50)**	0.002

* *p* < 0.05

** *p <* 0.01.

### 2.3 Cox Regression Analysis of MACE Risk Factors

(1) MACE was assessed as a binary endpoint during postoperative follow‐up (coded: 1 = occurred, 0 = did not occur); (2) In univariate analysis, GRACE score, lipoprotein(a), volume score, mass score, and Agatston score were all significantly associated with MACE occurrence (*p* < 0.05); (3) Multivariate Cox regression analysis revealed that elevated GRACE scores, Agatston scores, and lipoprotein(a) levels were independent risk factors for MACE occurrence after PCI in ACS patients (*p* < 0.05). See Table 3 at for details.

**Table 3:** Cox regression analysis results for MACE risk factors.

Variable	Training group				Test group			
	Univariate analysis		Multivariate analysis		Single‐factor analysis		Multifactor analysis	
	HR (95% CI)	P	HR (95% CI)	P	HR (95% CI)	P	HR (95% CI)	P
GRACE score	1.012	0.000	1.008	0.006	1.019	0.000	1.0	0.021
	(1.007–1.018)		(1.002–1.014)		(1.010–1.028)		(1.002–1.026)	
Volume Integration	1.002	0.000	0.997	0.101	1.002	0.001	1.001	0.459
	(1.001–1.002)		(0.993–1.001)		(1.001–1.004)		(0.999–1.003)	
Equivalent Mass	1.007	0.000	0.994	0.258	1.004	0.000	1.000	0.857
	(1.005–1.008)		(0.984–1.004)		(1.002 ~ 1.005)		(1.000–1.001)	
Agatston Score	1.002	0.000	1.005	0.012	1.003	0.000	1.002	0.039
	(1.001 ~ 1.002)		(1.001–1.009)		(1.002–1.004)		(1.000–1.004)	
Lipoprotein a	1.010	0.034	1.009	0.043	1.018	0.030	1.024	0.022
	(1.001–1.020)		(1.000–1.018)		(1.002–1.034)		(1.003–1.044)	

### 2.4 Combined Application Predictive Performance

**Figure 3:**
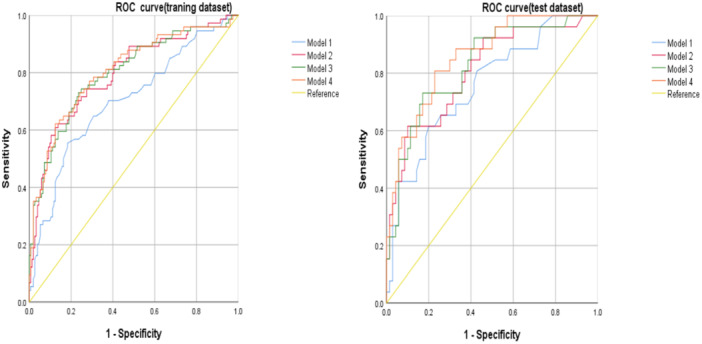
ROC curves for each model in the training set (left) and test set (right).

Compared to Model 1 [(AUC (95% CI) = 0.702 (0.627 ~ 0.777), AUC (95% CI) = 0.758 (0.650–0.865)]; Model 2 [(AUC (95% CI) = 0.795 (0.730–0.859), AUC (95% CI) = 0.808 (0.708 ~ 0.908)] demonstrated significant advantages. Combining clinical indicators with Agatston scores, the joint model exhibited superior predictive performance. Model 4 [(AUC (95% CI) = 0.806 (0.742 ~ 0.869), AUC (95% CI) = 0.857 (0.777 ~ 0.936)]. The sensitivity of the combined scores was 66.2% and 80.8%, while the specificity was 87.6% and 77.1% (Delong test, *p* < 0.01), as shown in Table 4 and Figure 3.

**Table 4:** ROC curve analysis results for GRACE score and Agatston score in predicting MACE.

Indicator	AUC (95% CI)		P		Sensitivity (%)		Specificity (%)	
	Training group	Test group	Training group	Test group	Training group	Test group	Training group	Test group
Model 1	0.702	0.758	0.000	0.000	82.0	80.0	55.0	62.0
GRACE Score	(0.627–0.777)	(0.650–0.865)						
Model 2	0.795	0.808	0.000	0.000	62.2	61.5	82.3	90.0
Agatston + lipoprotein a	(0.730–0.859)	(0.708–0.908)						
Model 3	0.798	0.827	0.000	0.000	73.0	73.1	76.5	82.9
GRACE Score + Agatston	(0.734–0.863)	(0.734–0.919)						
Model 4	0.806	0.857	0.000	0.000	62.2	80.8	87.6	77.1
GRACE Score + Agatston Score + Lipoprotein(a)	(0.742–0.869)	(0.777–0.936)						

### 2.5 Survival Curve Comparison of Composite Scores Across Groups

During follow‐up, 100 cases (30.86%) of MACE were recorded. According to GRACE risk stratification, 100 cases (30.86%) in the study cohort belonged to the high‐risk group, while 224 cases (69.14%) belonged to the non‐high‐risk group. Based on Agatston score risk stratification, the high‐risk group comprised 65 cases (20.06%), while the non‐high‐risk group included 259 cases (79.9%). Patients were further divided into four groups: Ga1 group: GRACE score ≥ 140, CACS score ≥ 400 (*n* = 36); Ga2 group: GRACE score < 140, CACS score ≥ 400 (*n* = 29); Ga3 group: GRACE score ≥ 140, CACS score < 400 (*n* = 71); Ga4 group: GRACE score < 140, CACS score < 400 (*n* = 188).

**Figure 4:**
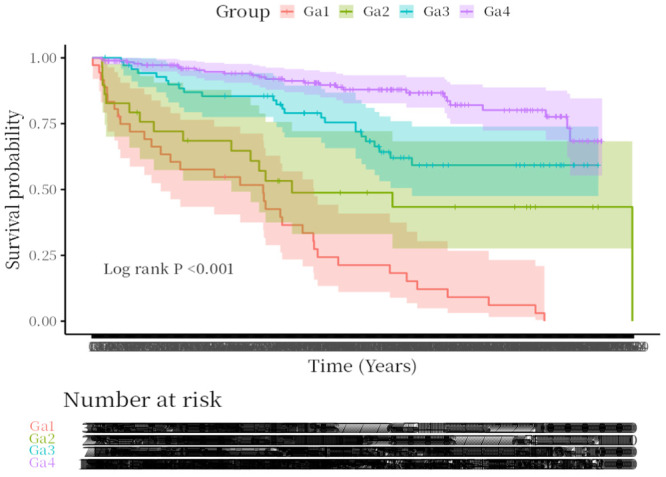
Survival curves for patients in each group.

Survival analysis revealed that MACE occurred in 34 patients (94.4%) in the Ga1 group, significantly higher than in the Ga2 group (16 cases, 55.17%), Ga3 group (24 cases, 33.80%), and Ga4 group (26 cases, 13.83%). The Log‐rank test (*p* < 0.001) demonstrated statistically significant differences in MACE incidence among the four groups, as shown in Figure 4.

### 2.6 Robustness Testing of Primary Conclusions: Sensitivity Analysis

To assess the robustness of our findings, we redefined the primary endpoint (MACE) to exclude “readmission due to heart failure” and reanalyzed the entire cohort. Results are presented in Table 5. Agatston score remained a strong independent predictor of MACE in the sensitivity analysis, with an adjusted hazard ratio (HR) of 1.002 (95% CI: 1.000–1.004, *p* = 0.022) under the new endpoint. This was highly consistent with the primary analysis result (HR = 1.002, 95% CI: 1.000–1.003, *p* = 0.021). The overall discriminatory power of the model was not substantially affected, as shown in Table 6., *p* = 0.021). The overall discriminatory ability of the model was not substantially affected, as shown in Table 6. The C‐statistic (AUC) for the full cohort in the model based on the new endpoint definition was 0.794 (95% CI: 0.740–0.848), comparable to the primary model′s 0.822 (95% CI: 0.772–0.872). The direction and strength of association for other major predictors did not change significantly.

**Table 5:** Comparison of multivariate Cox regression results between primary and sensitivity analyses.

	Primary analysis				Sensitivity analysis			
	Univariate analysis		Multivariate analysis		Single‐Factor analysis		Multivariate analysis	
Variable	HR (95% CI)	P	HR (95% CI)	P	HR (95% CI)	P	HR (95% CI)	P
GRACE score	1.014	0.000	1.010	0.000	1.012	0.000	1.008	0.002
	(1.010–1.019)		(1.005–1.015)		(1.007–1.017)		(1.003–1.013)	
Volume integral	1.002	0.000	1.000	0.814	1.002	0.000	0.999	0.371
	(1.002 ~ 1.002)		(0.998–1.001)		(1.001–1.002)		(0.997–1.001)	
Equivalent mass	1.004	0.000	1.000	0.906	1.003	0.000	0.999	0.457
	(1.003–1.005)		(0.997–1.003)		(1.002–1.004)		(0.996–1.002)	
Agatston Score	1.002	0.000	1.002	0.021	1.001	0.000	1.002	0.022
	(1.000 ~ 1.002)		(1.000–1.003)		(1.001–1.002)		(1.000–1.004)	
Lipoprotein a	1.013	0.002	1.012	0.004	1.011	0.011	1.009	0.033
	(1.005–1.021)		(1.004–1.020)		(1.002–1.019)		(1.001–1.017)	
High‐density lipoprotein cholesterol					0.713	0.078		
					(0.489–1.039)			

**Table 6:** Comparison of predictive model performance in primary and sensitivity analyses.

	Primary analysis		Sensitivity analysis	
Indicator	AUC (95% CI)	P	AUC (95% CI)	P
Model 1	0.714	0.000	0.690	0.000
GRACE Score	(0.651–0.776)		(0.626–0.755)	
Model 2	0.801	0.000	0.783	0.000
Agatston + lipoprotein a	(0.747–0.854)		(0.726–0.840)	
Model 3	0.806	0.000	0.781	0.000
GRACE Score + Agatston	(0.753–0.860)		(0.723–0.838)	
Model 4	0.822	0.000	0.794	0.000
GRACE Score + Agatston Score + Lipoprotein(a)	(0.772–0.872)		(0.740–0.848)	

## 3 Discussion

The readmission rate for ACS patients after coronary angiography has been increasing annually, impacting patient prognosis and quality of life. Therefore, we need to accurately identify high‐risk subgroups to guide personalized secondary prevention plans [16].

### 3.1 GRACE Score for Predicting MACE

The GRACE score was initially developed in 2001 for risk stratification in adults presenting with symptoms of acute coronary syndrome. Studies by Xiong S [17]. and others demonstrated that the GRACE score significantly improves predictive accuracy for MACE occurrence after PCI in ACS patients. This study, using Cox regression analysis, indicates that the admission GRACE risk score is an independent predictor of MACE occurrence, consistent with previous findings. However, other studies suggest that the GRACE score may overestimate risk in elderly patients presenting to the emergency department [18]. A recent cohort study demonstrated that incorporating certain biomarkers improves the risk stratification efficacy of this score; however, its AUC value remains below 0.8, indicating that the model′s performance requires further refinement [19].

### 3.2 Calcification Score for Predicting MACE

#### 3.2.1 Calculation Method

In 1990, Agatston [20] and colleagues pioneered the quantification of coronary artery calcification via cardiac CT scans, defining the Agatston score as calcified area (mm^2^) × density coefficient (based on CT values). Building upon the Agatston scoring method, Callister et al. [11] proposed a novel scoring approach—the volumetric scoring method—capable of accurately converting calcified lesions into volumetric measurements. Subsequently, in 2003, Hong et al. proposed a mass‐based scoring method, adjusting the calcium mass fraction based on the CT values of calcified plaques and converting it into equivalent calcium concentration [12, 21]. The “gold standard” for clinical risk assessment remains the Agatston score, while volume‐ and mass‐based scoring may offer advantages in specific scenarios.

#### 3.2.2 Advantages of Non‐Gated Chest CT Scanning

Traditional calcification scoring calculations routinely employ cardiac‐gated CCTA non‐contrast sequences, which are based on cardiac‐gated multislice spiral CT examinations. These sequences offer advantages such as non‐invasiveness and ease of operation [22, 23]. However, this approach significantly increases radiation exposure. Due to its high radiation dose and the requirement for contrast agent injection during examination, it presents substantial limitations for patients with radiation intolerance or renal failure who cannot tolerate contrast agents [24]. Therefore, this study employs a calcium score derived from non‐gated chest CT non‐contrast scans combined with the GRACE score to predict adverse cardiovascular events.

#### 3.2.3 Coronary Artery Calcification Score in Predicting MACE

A recent study found that among coronary artery disease patients undergoing PCI, the proportion of multivessel disease and calcification scores were significantly higher in the group with calcification scores exceeding 400 points compared to groups with CACS ≤ 100 points and CACS ≤ 400 points [13]. Coronary artery calcification score is an independent predictor of MACE after PCI in patients with angina pectoris [25]. In this study, univariate analysis showed that volumetric score, mass score, and Agatston score were all associated with MACE occurrence (*p* < 0.05). Notably, Yong Y [26] et al. also observed a negative correlation between coronary artery calcium density and cardiovascular events. However, due to limited sample size, this study failed to demonstrate a negative association between coronary artery calcium density and MACE after PCI in ACS patients. Multivariate Cox regression analysis revealed only the Agatston score as an independent risk factor for MACE after PCI in ACS patients (*p* < 0.05).

#### 3.2.4 GRACE Combined With Hemodynamic and Biochemical Markers Predicts MACE

Coronary artery inflammation has been associated with coronary atherosclerotic plaque instability, rupture, and myocardial infarction, as documented in relevant reports [27]. Studies confirm that combining the GRACE score with inflammatory markers such as the ratio of white blood cells to mean platelet volume and the ratio of monocytes to high‐density lipoprotein cholesterol provides incremental predictive value for major endpoints ^beyond^the GRACE risk score ^alone^ [28, 29]. In recent years, biomarkers have gained widespread recognition for their value in predicting outcomes of acute myocardial infarction. Among these, N‐terminal pro‐brain natriuretic peptide (NT‐proBNP) serves as a common indicator for assessing myocardial injury, while lipoprotein‐associated phospholipase A2 (Lp‐PLA2) represents a novel inflammatory marker whose serum levels can be utilized for risk stratification in acute coronary syndrome [30]. Patients with MACE following PCI for acute myocardial infarction exhibited abnormally elevated GRACE scores and serum NT‐proBNP and Lp‐PLA2 levels. Consistent with our findings, lipoprotein(a) is an independent predictor of MACE and provides incremental value to GRACE scoring.

#### 3.2.5 GRACE Combined With Calcification Score Predicts MACE

Yao X et al. [31] found a positive correlation between coronary artery calcification scores and GRACE scores. Therefore, this study aimed to evaluate the predictive value of combining coronary artery calcification scores with GRACE scores for adverse cardiovascular events after PCI in ACS patients. Results showed that the combined score provided superior predictive performance compared to the GRACE score alone. In this study, survival curves were plotted and compared across four patient groups based on risk‐stratified grouping using the two composite scores, revealing statistically significant differences. This further confirms that the combined GRACE and calcification score model provides robust support for predicting outcomes in ACS patients after PCI. It is important to note that patients with moderate GRACE scores but high CAC may require closer follow‐up.

### 3.3 Limitations

Our study has certain limitations. First, this is a single‐center, retrospective study with a small sample size and lacks an external validation cohort. Second, CT scans were performed using multiple scanning devices; we minimized variability through standardized scanning protocols and strict quality control. Third, the retrospective design carries inherent risks of selection bias. To mitigate this, we employed a continuous enrollment approach to ensure all eligible patients within the study period were included, thereby minimizing artificial selection.

## 4 Conclusion

In summary, coronary artery calcium scoring obtained from non‐gated chest CT demonstrates significant incremental value compared to GRACE score in predicting adverse cardiovascular events. The combination of these two indices enables a comprehensive assessment of acute event risk and chronic atherosclerotic burden. Therefore, suggests that using a combined score (GRACE + CACS) in follow‐up management after PCI may add value to risk restratification.

## Author Contributions

**Zhaoyuan Xing:** software, methodology, writing – original draft, formal analysis, resources, supervision, visualization, project administration, validation, data curation, investigation. **Haoyan Pan:** writing – original draft. **Huan Ding:** writing – original draft. **Jianing Chen:** writing – original draft. **ZiGuang Huang:** writing – original draft. **Jing Wen:** writing – review and editing. **Zhe Zhang:** writing – review and editing. **Baoying Zhao:** writing – review and editing, resources, methodology. **Xu Dai:** funding acquisition, supervision, investigation, writing – review and editing, software, writing – original draft, resources, data curation, validation, project administration, visualization, conceptualization, formal analysis.

## Funding

The authors received no specific funding for this work.

## Ethics Statement

All procedures performed in studies involving human participants were in accordance with the ethical standards of the institutional and/or national research committee and with the 1964 Helsinki declaration and its later amendments or comparable ethical standards. This study was approved by the Medical Ethics Committee of Liaoning University of Traditional Chinese Medicine, (Review Opinion No. Y2025005CS(KT)‐005‐01).

## Consent

Consent for publication was obtained for every individual person′s data included in the study.

## Conflicts of Interest

The authors declare no conflicts of interest.

## Data Availability

The datasets generated during and/or analyzed during the current study are available from the corresponding author upon reasonable request for the purpose of validating the research findings. However, the data are not publicly available due to privacy or ethical restrictions.
